# CD154 inhibits death of T cells via a Cis interaction with the α5β1 integrin

**DOI:** 10.1371/journal.pone.0235753

**Published:** 2020-08-03

**Authors:** Meriem Bachsais, Suzanne Salti, Kossay Zaoui, Ghada S. Hassan, Fawzi Aoudjit, Walid Mourad

**Affiliations:** 1 Laboratoire d’Immunologie Cellulaire et Moléculaire, Centre de Recherche du Centre Hospitalier de l’Université de Montréal (CR-CHUM), Montréal, Québec, Canada; 2 Centre de recherche du CHU de Québec-Université Laval, Québec, Québec, Canada; Thomas Jefferson University, UNITED STATES

## Abstract

CD154 plays a major role in the pathogenesis of several autoimmune and inflammatory diseases. In addition to CD40, soluble CD154 (sCD154) binds to other receptors namely αIIbβ3, αMβ2, α5β1 and αvβ3 integrins. We have previously reported that binding of sCD154 to α5β1 integrin expressed on several human T cell lines is capable of inhibiting Fas-induced cell death. In the current study, we show that such effect of the sCD154/α5β1 interaction is not restricted to the cell death response induced by Fas but could also be exhibited toward other death signals such as TRAIL and TNF- α. We also demonstrate that sCD154 is capable of inhibiting Fas-mediated death of human activated T cells, more importantly of CD4^+^ than CD8^+^ T ones. Our data also show that membrane-bound CD154 and α5β1 integrin expressed on the surface of distinct cells failed to influence cell death responses. However, when membrane-bound CD154 and α5β1 are expressed on the surface of same cell, their interaction was capable of down regulating cell death. CD154 was shown to co-localize with the α5β1 integrin on the surface of these cells. These data strongly suggest a cis-type of interaction between CD154 and α5β1 when both are expressed on the same cell surface, rather than a trans-interaction which usually implicates the ligand and its receptor each expressed on the surface of a distinct cell. Taken together, these findings add to the list of roles through which CD154 is contributing to the pathogenesis of autoimmune-inflammatory diseases, i.e. by protecting T cells from death and enhancing their survival.

## Introduction

CD154, also known as CD40 ligand (CD40L), is an immunomodulator initially described in activated CD4-positive T cells and later found to be expressed on other types of cells such as basophiles, mast cells, activated CD8-positive T cells and platelets [1, 2]. Similarly to other members of the TNF family, in addition to the membrane-bound molecule, CD154 also exists in a soluble form (sCD154) that is still biologically active [3]. This soluble form is usually released from activated T cells and platelets by proteolytic cleavage [3, 4]. Soluble CD154 is exhibited at high levels in many inflammatory disorders [5–7], including rheumatoid arthritis (RA) and sytemic lupus erythromatus (SLE) diseases [8, 9].

Together with its classical receptor CD40, CD154 is implicated in humoral as well as cell-mediated immunity [2, 10]. By acting on several immune/inflammatory cells, CD154 influences their functions and activation status [2, 11]. Interestingly, during cell/cell interaction, binding of CD154 to CD40 molecules leads to bidirectional signals that modulate cell functions [12–15]. Blocking the CD154/CD40 interaction using different experimental approaches was shown to completely abolish the development of several autoimmune conditions [2, 16], such as RA and SLE [17–20].

In addition to CD40, sCD154 was shown to bind other receptors, namely the αIIbβ3 [21], αMβ2 (Mac-1) [22], α5β1 [23] and αvβ3 integrins [24]. The interaction of sCD154 with αIIbβ3 on platelets was shown to stabilize thrombus under high sheer conditions [25], while that with αMβ2 was reported to promote the development of inflammation in the vessels and atherosclerosis [22], and to play a role in Th1 immune responses against *Leishmania major* infections [26]. The αvβ3 integrin was identified as a receptor for CD154. Although no functional studies were undertaken but authors expected a high biological significance for the CD154/αvβ3 interaction given the high expression of αvβ3 on vascular and cancer cells [24].

In this context, we have demonstrated that stimulating an α5β1-positive monocytic cell line with sCD154 induces the activation of MAPK/ERK1/2 pathway and IL-8 production in a CD40-independent manner [23]. Interestingly, ligation of the α5β1 integrin simultaneously with ligation of CD40 was shown to activate p38 and ERK1/2 MAPK and to synergize in the release of inflammatory mediators such as MMP-2 and -9 [27]. Furthermore, the physiopathological relevance of the CD154/α5β1 dyad could be implicated in the development of allergic asthma given data showing that the CD154/α5β1 interaction enhances the production of inflammatory cytokines in T cells and bronchial fibroblasts of asthmatic patients during cell/cell interaction [28]. Interestingly, our recent results showed that CD154 is capable of binding to several T cell lines via their α5β1 integrin inducing the activation of p38, ERK, and Akt [29]. We also demonstrated that treatment of these cells with CD154 entirely abrogated their Fas-induced death, in a mechanism involving activation of PI-3K and a decreased cleavage of caspase-8 [29].

Given that T cell survival and persistence is a characteristic signature of numerous inflammatory and autoimmune diseases, we aimed herein at further examining the effect of the CD154/α5β1 dyad on T cell survival. We found that binding of sCD154 to α5β1 integrin also inhibits apoptosis of T cell lines and human primary T cells induced by the TNF-related apoptosis-inducing ligand (TRAIL) and TNF-α. Furthermore, our studies suggest that the anti-apoptotic effect of CD154 is exerted in a cis-dependent manner, i.e both CD154 and α5β1 are expressed on the surface of the same cell.

## Materials and methods

### Ethics statement

This study is using PBMC samples from healthy subjects. This study received approval from the Research Center of the Centre Hospitalier de l’Université de Montréal (CRCHUM) ethical committee (no: SL-06.077). All human subjects that donated biological samples for this study provided written informed consent for their participation.

### Reagents and antibodies

Recombinant soluble CD154 (sCD154) was generated as previously described [14]. Anti-CD95 or anti-Fas (clone CH-11, an IgM) was purchased from Chemicon (Temecula, CA). Recombinant human TRAIL (rhTRAIL) and rhTNF were obtained from R&D Systems (Minneapolis, USA). Anti-CD95 (LOB3/17, an IgG) was purchased from Bio-Rad Serotec Inc. (Raleigh, NC). The hybridoma producing antibodies raised against human CD154 (mAb C4.14) was produced in our laboratory. Anti-CD154 hybridoma 5C8 (IgG_2a_) and anti-CD40 hybridoma G28.5 (IgG_1_) were obtained from ATCC. Anti-αMβ2 antibodies (clone ICRF44) were obtained from BD Biosciences (Mississauga, ON). Anti-α5β1 (clone JBS5) and anti-αIIbβ3 (clone A2A9/6) were purchased from Santa Cruz Biotechnology (Santa Cruz, CA). The anti-β1 mAb (clones B44) was a generous gift from Dr. John A. Wilkins (Manitoba Centre for Proteomics and Rheumatic Diseases, University of Manitoba, Winnipeg, MB). The anti-TNF-R1 (clone H398), anti-TRAIL-R1 (clone 69036) and anti-TRAIL-R2 (clone 71908) antibodies were purchased from R&D Systems (Minneapolis, USA). The GAM-alexa488 was obtained from Invitrogen Life Technology (Burlington, Ontario, Canada). The biotin-labelled CD40-Fc was prepared using the method provided by Pierce (Rockford, IL, USA).

### Cell lines

The human Jurkat E6.1 T cell line, the BJAB B lymphoma cell line and the embryonic kidney cell line HEK 293 were obtained from ATCC (Manassas, VA) and maintained at 37°C in RPMI 1640 media supplemented with 5% fetal bovine serum (FBS), 1% L-glutamine, penicillin, streptomycin (GIBCO, Burlington, ON, Canada). Jurkat E6.1 and HEK 293 were stably transfected as we previously described [30] with human CD154 (hCD154) or empty vector (vect) and were maintained at 37°C in DMEM media supplemented with 5% FBS, 1% L-glutamine, penicillin, streptomycin and 100·g/ml zeomycin (Invivo Gen, Cederlane Laboratories, Burlington, Canada).

### Isolation of human T cells and their activation

Peripheral blood mononuclear cells (PBMC) of healthy donors were isolated by Ficoll–Hypaque density gradient centrifugation. Purified CD3^+^, CD4^+^ and CD8^+^ T cells were isolated from PBMC by negative selection using magnetic beads (STEMCELL Technologies). The purity of the isolated CD3^+^ CD4^+^ and CD8^+^ T cells was > 96%, as determined by flow cytometric analysis. Isolated cells were then cultured in RPMI 1640 media supplemented with 10% fetal bovine serum (FBS) and 1% L-glutamine, penicillin and streptomycin and cultured in the presence of PHA (5 ug/ml) (Sigma-Aldrich, Oakville, Ontario, Canada) and IL-2 (20 U/ml) (R&D Systems) for a period of six days.

### Flow cytometry analysis

The cell surface expression of CD154, CD40, α5β1, αMβ2, αIIbβ3, Fas, TNFR1, TRAILR1 and TRAILR2 was determined by FACS analysis. Cells were stained with the appropriate control isotype IgGs or respective antibodies (anti-CD154 mAb, C4; anti-CD40 mAb, G28.5; anti- α5β1 (active form) mAb, B44; anti-α5β1 (inactive form) mAb, JBS5; anti-αMβ2 mAb, ICRF44; anti-αIIbβ3 mAb, A2A9/6; anti-CD95 mAb, LOB3/17; anti-TNF-R1 mAb, H398; anti-TRAIL-R1 mAb,C69036 and anti-TRAIL-R2 mAb, C71908, followed by goat anti-mouse IgG-Alexa488 staining. Samples were analysed on FACSCalibur flow cytometer (BD Biosciences, Mississauga, Ontario, Canada).

### CFSE staining fluorescence microscopy

Jurkat E6.1 and BJAB cells were suspended at 10 million/ml in PBS. CFSE (Life Technologies, Ontario, Canada) was added to a final concentration of 1uM for 10 min at 37° in a water bath. After adding the RPMI 10% FBS 10min at room temperature, cells were centrifuged then suspended in RPMI at the desired concentration before use. Labelled Jurkat E6.1 and BJAB cells were added on HEK 293-vect. or HEK 293-hCD154 already adhered to 96 well plates. After 5 min at 37°C, wells were washed to remove the free Jurkat E6.1 and BJAB cells. In some conditions, co-cultures were treated with 5C8 mAb to block the CD40/CD154 interaction. Cell/cell interactions mediated by CD154/α5β1 and CD154/CD40 were analyzed by fluorescence microscopy.

### Immunofluorescence microscopy

CD154-transfected Jurkat E6.1 cells and non-transfected ones were grown on coverslips, fixed in 4% formaldehyde (MilliporeSigma, Billerica, MA, USA) and permeabilized in 0.2% Triton X-100 (ThermoFisher Scientific, Ontario, Canada). To detect CD154 molecules, biotinylated sCD40-Fc was added followed by streptavidin PE, whereas detection of α5β1 integrin was undertaken by adding JBS5 mAb followed by GAM-alexa488. DNA was counterstained with Hoechst dye (ProLong^™^ Gold Antifade Mountant with DAPI (Thermo Fisher Scientific). Images were recorded using a microscope (ApoTome.2, Observer. Z1; Carl Zeiss, Inc.) with a 63× plan Apo 1.4 NA objective coupled to a camera (AxioCamHRm; Carl Zeiss, Inc.) and driven by AxioVision LE software (Carl Zeiss, Inc.). The degree of co-localization, expressed as the Pearson’s correlation coefficient (proportion of all red intensities that have green components among all red intensities), was assessed by the co-localization analysis function of Imaris software (Bitplane^®^). Results were logged into Excel Microsoft Excel^®^ for analysis. All values are means ± SD from 16 cells.

### Cell death

Cells were cultured in the presence or absence of sCD154 for 6 hrs and then treated with anti-Fas, rhTNF-α, rhTRAIL for an additional 18 hours at 37°C. In some experiments and when indicate, Wortmannin (Millipore Sigma, Bilerica, MA, USA) was used as a control. Cell death was evaluated by FACS analysis as the percentage of death obtained by propidium iodide (Invitrogen, Burlington, ON) staining as we previously described [29].

### Statistical analysis

Data will be analyzed by one-way ANOVA using Tukey’s multiple comparison test or by the Student’s t test (for simple comparisons).

## Results

### 1-Soluble CD154 inhibits TRAIL- and TNF-α-induced cell death in Jurkat E6.1 T cells

**Fig 1:**
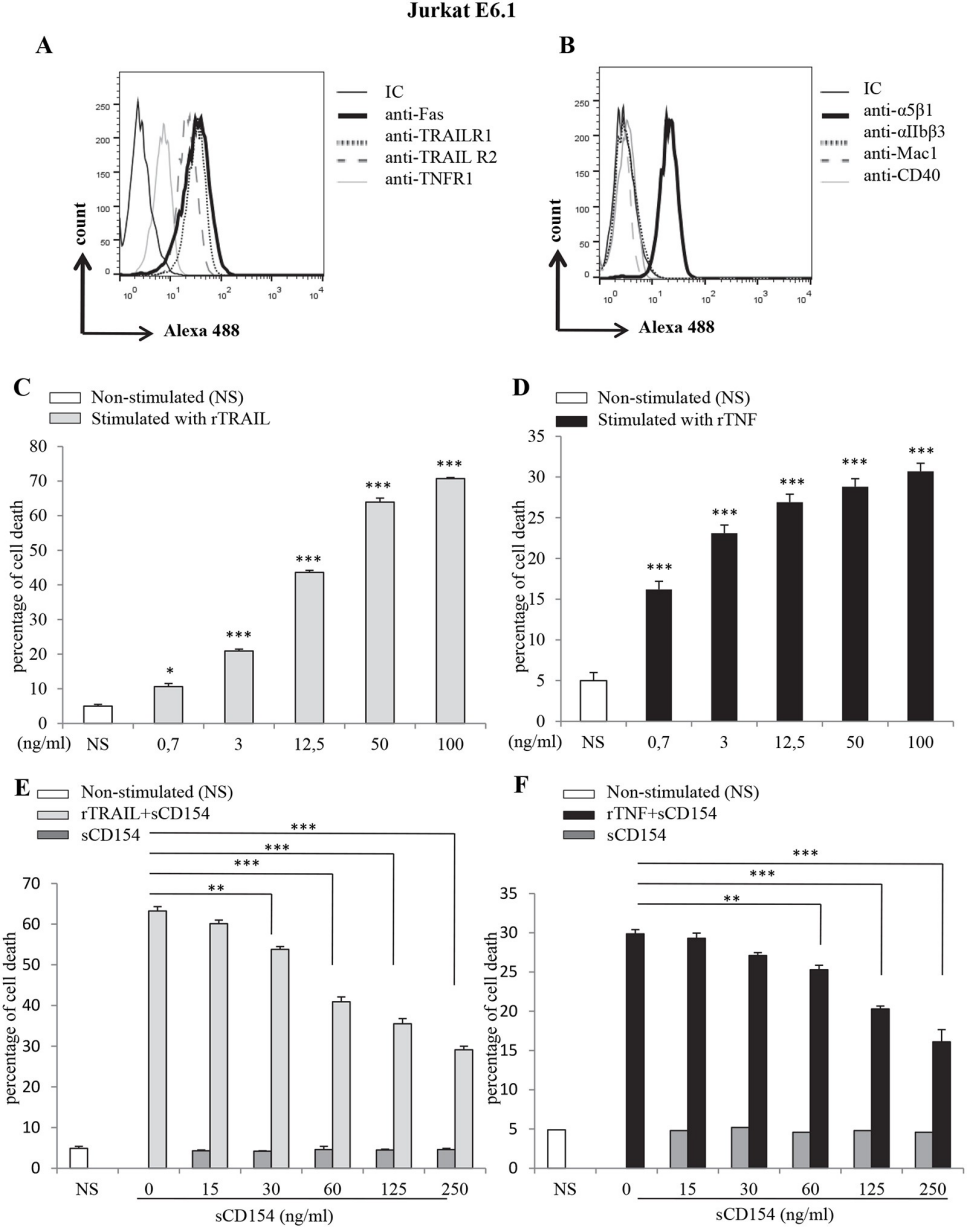
Soluble CD154 inhibits TRAIL- and TNF-α-mediated cell death in Jurkat E6.1 cells. *(A*, *B)* Cells were incubated with anti-TNF-R1 mAb (H398), anti-TRAIL-R1 mAb (C69036), anti-TRAIL-R2 mAb (C71908), anti-α5β1 integrin mAb (JBS5), anti-αMβ2 mAb (ICRF44), anti-αIIbβ3 mAb (A2A9/6), anti-CD40 mAb (G28.5) or isotype IgGs followed by goat anti-mouse IgG-Alexa488. The expression of the corresponding molecule on cell surface was analysed by FACS Calibur. *(C*, *D) Cell death response of Jurkat E6*.*1 cells stimulated with TRAIL or TNF-α*. Jurkat E6.1 T cells were stimulated or not (NS) with different concentrations of recombinant TRAIL (rTRAIL) *(C)* or rTNF-α *(D)* for 18 hours at 37°C and assessed for their cell death response by FACS analysis as the percentage of death obtained by propidium iodide staining. Statistical testing compared cells stimulated with rTRAIL or rTNF-α with NS cells. *(E*, *F) Soluble CD154 inhibition of Jurkat T cell death*. T cells were pre-treated with different concentrations of sCD154 for 6h, then left non-stimulated (NS) or stimulated with sub-optimal concentration of rTRAIL (50 ng/ml) *(E)* or rTNF-α (12ng/ml) *(F)* for 18 hours at 37°C and assessed for their cell death response by FACS analysis. Results represent mean values ±SD and are representative of at least 5 independent experiments (** p < 0.*01*, *** p < 0.*001*).

We have previously reported that binding of sCD154 to several human T cell lines expressing α5β1 molecules inhibits their Fas-induced death [29]. We therefore hypothesized that such dyad plays an important role in the modulation of T cell death induced by other apoptotic stimuli. As Jurkat E6.1 cells are also susceptible to death induced by other members of the TNF family, namely TRAIL and TNF-α [31–33], we aim here at assessing the impact of sCD154 on death induced by TRAIL and TNF-α in Jurkat E6.1 cells. In the first set of experiments, we analyzed the expression of the different death receptors and the CD154 receptors on the surface of these cells. Our results show that Jurkat E6.1 cells express TRAIL-R1, TRAIL-R2 and TNF-R1 on their surface (Fig 1A), and only the α5β1 integrin as the CD154 receptor (Fig 1B). It is worth noting here and as previously reported, that Jurkat E6.1 cells express only the inactive form of α5β1, capable of binding CD154 [23], and that binding of sCD154 to these cells was completely abolished by soluble α5β1 confirming the specificity of sCD154 to α5β1 integrin [29].

Given these data, we next evaluated the death response induced by different concentrations of human recombinant TRAIL (rTRAIL) and rTNF-α in Jurkat E6.1 cells. Our data show that treatment with rTRAIL or rTNF induces an apoptotic effect in Jurkat E6.1 cells in a concentration-dependent manner (Fig 1C). This cell death response reached its maximal level at around 100 ng/ml of TRAIL and at 50 ng/ml of TNF-α. We next investigated the capacity of sCD154 to inhibit these death responses. Cells were pre-incubated with different concentrations of sCD154 and then treated with the sub-optimum apoptosis-inducing concentration of TRAIL (50 ng/ml) or TNF-α (25 ng/ml) outlined above. Our data show that CD154 was capable of inhibiting TRAIL- and TNF-induced cell death in a dose-dependent manner (Fig 1D), as it did with Fas-induced response previously described [29]. It is important to mention here that we have shown before the capacity of agonistic anti-α5β1Abs to inhibit Fas-induced cell death in Jurkat E6.1cells as does CD154 [29], highly supporting the role of α5β1 integrin in the apoptosis-inhibiting effect of CD154 in Jurkat E6.1 cells. Another point to mention and referring to the same study, our results have previously shown that CD154 is capable of abrogating Fas-induced cell death in Jurkat E6.1 cells by inhibiting their caspase-8 cleavage [29], highly suggesting that a similar mechanism is implicated in the apoptosis-inhibiting effect of CD154 observed in our current study. Together, these results demonstrate that the CD154/α5β1 interaction inhibits TRAIL and TNF-α-induced death in T cell lines, confirming a role of this dyad in the persistence of T cells at the site of inflammation [34–36].

### 2-Soluble CD154 influences Fas-induced cell death in activated human CD3-positive T cells

**Fig 2:**
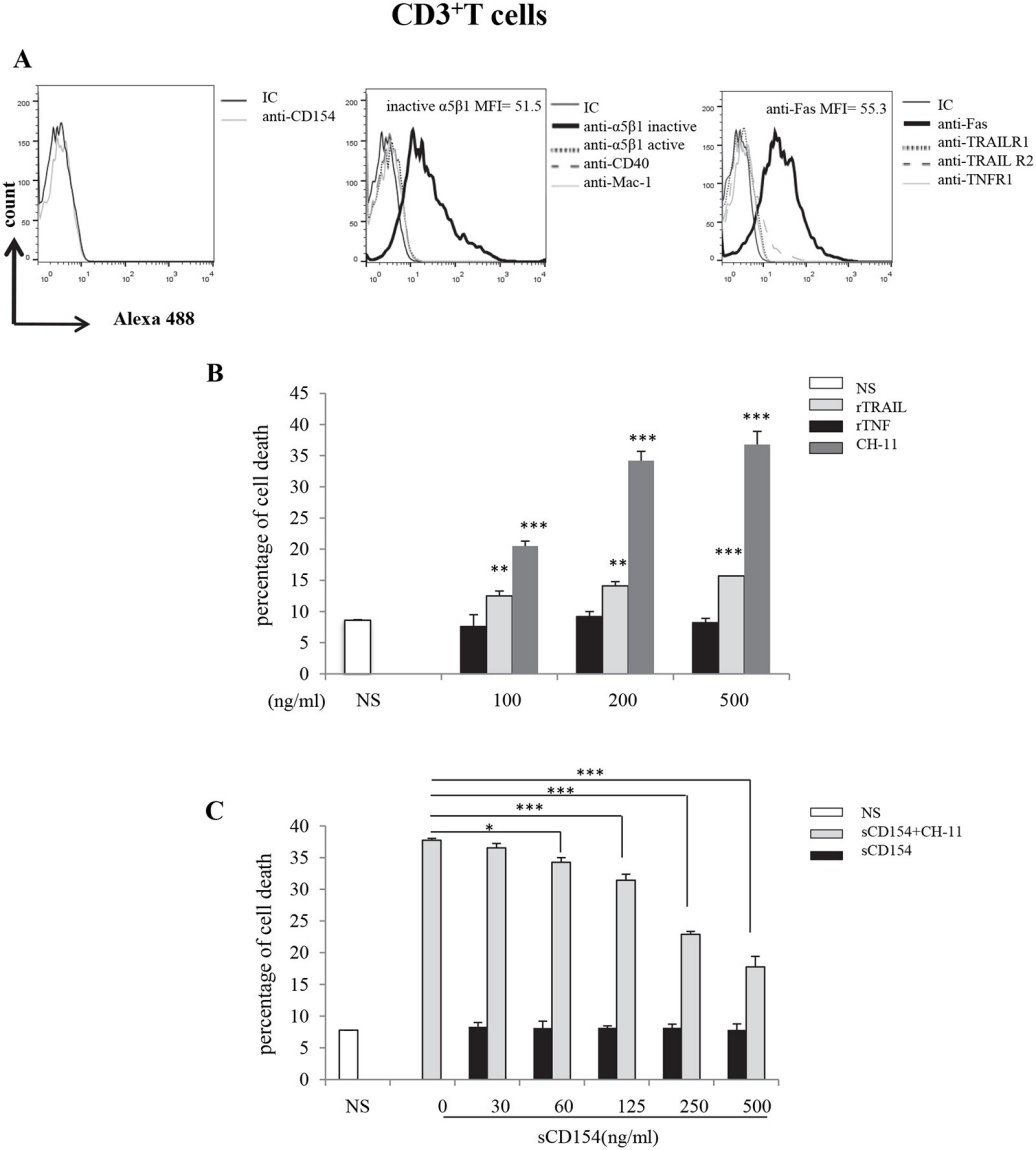
Soluble CD154 inhibits Fas-mediated cell death in human activated CD3^+^ T cells. *(A) Expression of α5β1*, *CD40*, *Mac-1*, *Fas and TRAIL and TNF receptors in human activated T cells*. T cells were isolated into CD3^+^ cells and activated with PHA/IL-2 for 6 days. Cells were then incubated with anti-CD154 (C4) *(left panel)*, anti-α5β1 (JBS5 and B44), anti-CD40 (G28.5) and anti-Mac-1(ICRF44) *(middle panel)*, and anti-Fas (LOB3/17), anti-TRAIL-R1 (C69036), anti-TRAIL-R2 (C71908) and anti-TNF-R1 (H398) *(right panel)* mAbs or the corresponding isotype IgGs followed by goat anti-mouse IgG-Alexa488 staining and evaluated for expression of the corresponding molecule on cell surface by FACS analysis. MFI = mean fluorescence intensity. *(B) Cell death response of CD3*^+^ *T cells stimulated with anti-Fas*, *TRAIL or TNF*. Activated CD3^+^ T cells were left non-stimulated (NS) or stimulated with different concentrations of rTRAIL, rTNF or CH-11 (anti-Fas) for 24 hours at 37°C and assessed for cell death response by FACS analysis as the percentage of death obtained by propidium iodide staining. Statistical testing compared cells stimulated with rTRAIL, rTNF-α or CH-11 with NS cells. *(C) Soluble CD154 inhibition of Fas-induced human CD3*^+^ *T cell death*. Activated CD3^+^ T cells were pre-treated with different concentrations of sCD154 for 6h, then left non-stimulated (NS) or stimulated with the CH-11 mAb (200ng/ml) for 24h at 37°C. Cell death was then evaluated using propidium iodide by FACS. Results represent mean values ±SD and are representative of 5 independent experiments performed with T cells isolated from 5 different blood donors (* *p < 0*.*05*, ** p < 0.*01*, *** p < 0.*001*).

Having demonstrated the role of sCD154 in inhibiting cell death induced by various death signals in T cell lines, it was crucial to investigate the influence of CD154 on the apoptotic response of primary T cells. PBMCs from the blood of healthy donors were purified and CD3^+^ T cells were isolated. Cells were then cultured in the presence of a combination of PHA and IL-2 [37] for a period of six days, to ensure their susceptibility to Fas-induced cell death as well as the release of all CD154 from their cell surface. We first analyzed the expression of CD154 and its receptors and that of various death receptors on the surface of these activated T cells. Our data show that human T cells and as described previously [38], lose their CD154 expression after activation (Fig 2A, left panel). Of the CD154 receptors, T cells only express the α5β1 integrin (Fig 2A, middle panel). The expression of α5β1 is heterogeneous in CD3^+^ T cells. It is worth mentioning here that we did not account for the expression of αIIbβ3 on T cells, as this integrin is exclusively expressed on platelets and on megakaryocytes in bone marrow [39]. The Fas molecule is the only cell death receptor detected on activated T cells (Fig 2A, right panel).

We next evaluated the death response induced via the Fas receptor and those of TRAIL and TNF-α in activated T cells. Cells were treated with different concentrations of CH-11 mAb, rhTRAIL or rhTNF-α for 24 hrs. Our data show that treatment of CD3^+^ T cells with CH-11 mAb was capable of inducing their apoptosis in a concentration-dependent manner (Fig 2B). This cell death response reached a steady state at around 200 ng/ml of CH-11. On the other hand, CD3^+^ cells showed no death susceptibility to TNF-α and a slight one to TRAIL at all concentrations tested (Fig 2B). Similarly to the protocol undertook with Jurkat E6.1 cells above, we subsequently assessed the Fas-induced apoptosis in activated CD3^+^ T cells in the presence of different concentrations of sCD154. Our data show that pre-incubation of activated T cells with sCD154 inhibited Fas-mediated apoptosis in a dose-dependent manner (Fig 2C).

### 3-Soluble CD154 inhibits Fas-mediated cell death more importantly in activated CD4^+^ T cells than CD8^+^ ones

**Fig 3:**
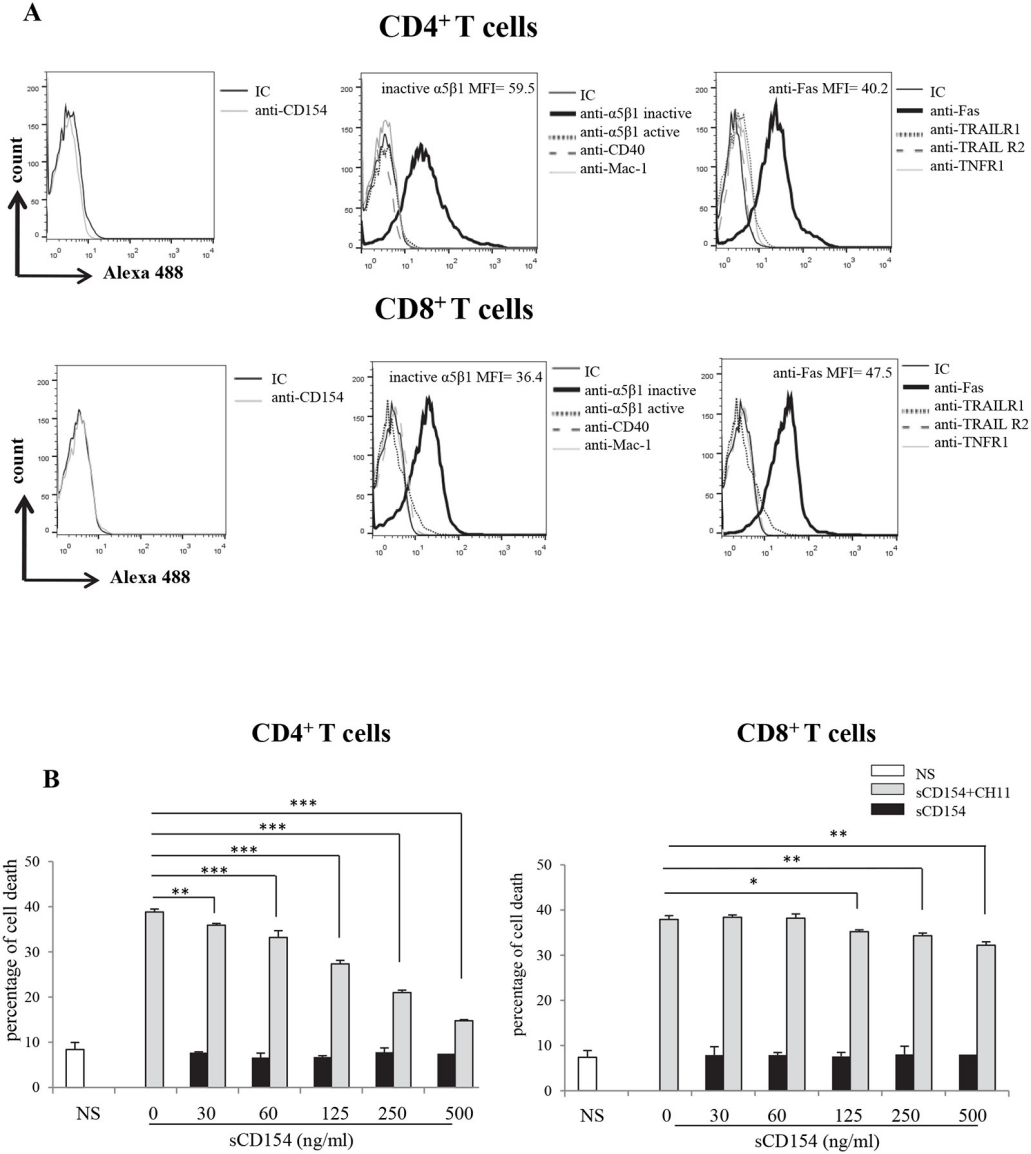
Soluble CD154 inhibits Fas-mediated cell death more importantly in human activated CD4^+^ T cells than CD8^+^ ones. *(A) Expression of α5β1*, *CD40*, *Mac-1*, *Fas*, *TRAIL and TNF receptors in human activated CD4*^+^ *and CD8*^+^ *T cells*. T cells were isolated by negative selection into CD4^+^ T cells and activated with PHA/IL-2 for 6 days. Cells were then incubated with anti-CD154 (C4) *(left panels)*, anti-α5β1 (JBS5 and B44), anti-CD40 (G28.5) and anti-Mac-1 (ICRF44) *(middle panels)*, and anti-Fas (LOB3/17), anti-TRAIL-R1 (C69036), anti-TRAIL-R2 (C71908) and anti-TNF-R1 (H398) *(right panels)* mAbs or the corresponding isotype IgGs followed by goat anti-mouse IgG-Alexa488 staining and evaluated for expression of the corresponding molecule on cell surface by FACS analysis. MFI = mean fluorescence intensity. *(B) Soluble CD154 inhibition of Fas-induced- CD4*^+^ *and -CD8*^+^ *T cell death*. Activated CD4^+^ and CD8^+^ T cells were pre-treated with different concentrations of sCD154 for 6h, then left non-stimulated (NS) or stimulated with the CH-11 mAb for 24h at 37°C. Cell death was then evaluated using propidium iodide by FACS. Results represent mean values ±SD and are representative of 5 independent experiments performed with T cells isolated from 5 different blood donors (* *p < 0*.*05*, ** p < 0.*01*, *** p < 0.*001*).

Based on the above observation showing the heterogeneous expression of α5β1 integrin in CD3^+^ T cells, PBMCs were purified into CD4^+^ and CD8^+^ T cells. Cells were activated with PHA and IL-2, as described above for a period of six days, and assessed for the expression of CD154 and its receptors and that of various death receptors on their surface. Our data show that CD4^+^ cells express a higher level of the α5β1 integrin in comparison with CD8^+^ T cells. As to the death receptors, both T cell populations express only the Fas receptor and at comparable levels (Fig 3A).

As we did with CD3^+^ T cells, we evaluated the death response induced via the Fas receptor in activated CD4^+^ and CD8^+^ T cells in the presence of different concentrations of sCD154. Our data show that pre-incubation of these activated T cell populations with sCD154 inhibited Fas-mediated apoptosis in a dose-dependent manner (Fig 3B). Such protective effect of CD154 against Fas-mediated cell death was more importantly exhibited in CD4^+^ cells as compared to CD8^+^ ones. Indeed, CD154 slightly inhibited the Fas-induced apoptosis in CD8^+^ cells and only when used at a relatively high concentration (Fig 3B).

Together, these results demonstrate that the CD154/α5β1 interaction inhibits the Fas-induced death of human T cells, more importantly that of the CD4^+^ subpopulation, reflecting a role of this dyad in the survival and persistence of T cells in inflammatory diseases.

### 4- Membrane-bound CD154 expressed on HEK 293 failed to influence the Fas-mediated cell death of Jurkat E6.1 and activated CD3-positive T cells

**Fig 4:**
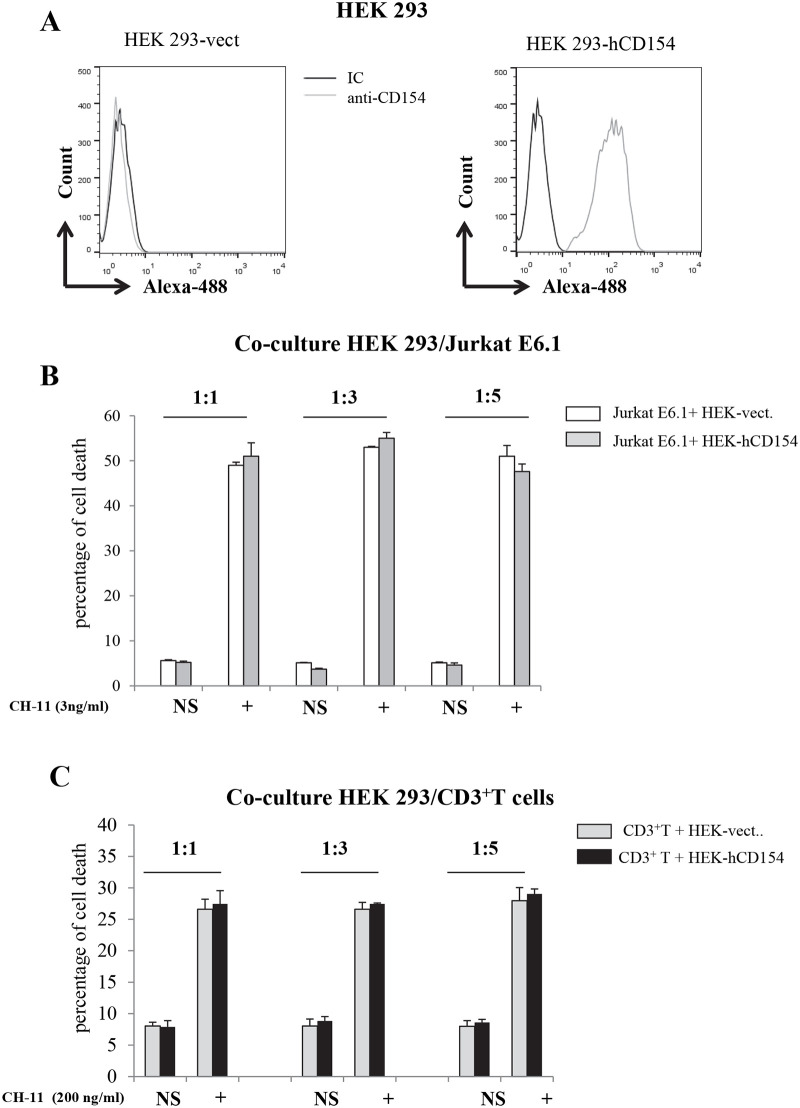
Interaction of membrane-bound CD154 with integrin α5β1 does not inhibit Fas-mediated cell death in Jurkat E6.1 cells and human T cells. *(A)* HEK 293 cells were stably transfected with human CD154 (HEK 293-hCD154) or empty vector (HEK 293-vect.). Cells were incubated with anti-CD154 C4 or the corresponding isotype IgGs followed by goat anti-mouse IgG-Alexa488 staining and evaluated for CD154 expression on cell surface by FACS analysis. *(B-C) Cell death response of Jurkat E6*.*1 cells and human T cells stimulated with Fas ligand in the presence or absence of membrane-bound CD154*. *(B)* Jurkat E6.1-NT cells and *(C)* human T cells were co-cultured with HEK 293-vect. or HEK 293-hCD154 using different cell/cell ratios (1:1, 1:3 and 1:5) for 6h at 37°C and stimulated with constant concentrations of anti-CH-11 (3ng/ml or 200ng/ml respectively) for 18 hours at 37°C. Cell death response was determined by FACS analysis as the percentage of death obtained by propidium iodide staining. Results represent mean values ±SD and are representative of at least 5 independent experiments.

It is well established that membrane-bound CD154 induces a stronger response in CD40- expressing cells than sCD154 [40, 41]. We therefore assessed the ability of membrane-bound CD154 to elicit similar and/or higher responses in cells expressing membrane-bound α5β1 with regard to Fas-induced cell death. For this purpose, we used Jurkat E6.1 and activated CD3-positive T cells as the α5β1 positive/CD154 negative cells and the HEK 293 transfected with CD154, as the CD154-positive cells (or the non-transfected ones as the negative control). Data in Fig 4A shows the expression of CD154 on the surface of the CD154-transfected HEK 293 cells. We co-cultured Jurkat E6.1 or activated CD3-positive T cells with adherent HEK 293 transfected or not with CD154 at different cell/cell ratios (1:5, 1:3, and 1:1), and stimulated these cells with the death ligand for Fas receptor. Surprisingly, our data show that Jurkat E6.1 and activated CD3-positive T cells co-cultured with HEK 293 cells transfected with CD154 exhibited similar death levels to those cultured in the presence of non-transfected CD154-negative HEK 293 cells (Fig 4B and 4C).

**Fig 5:**
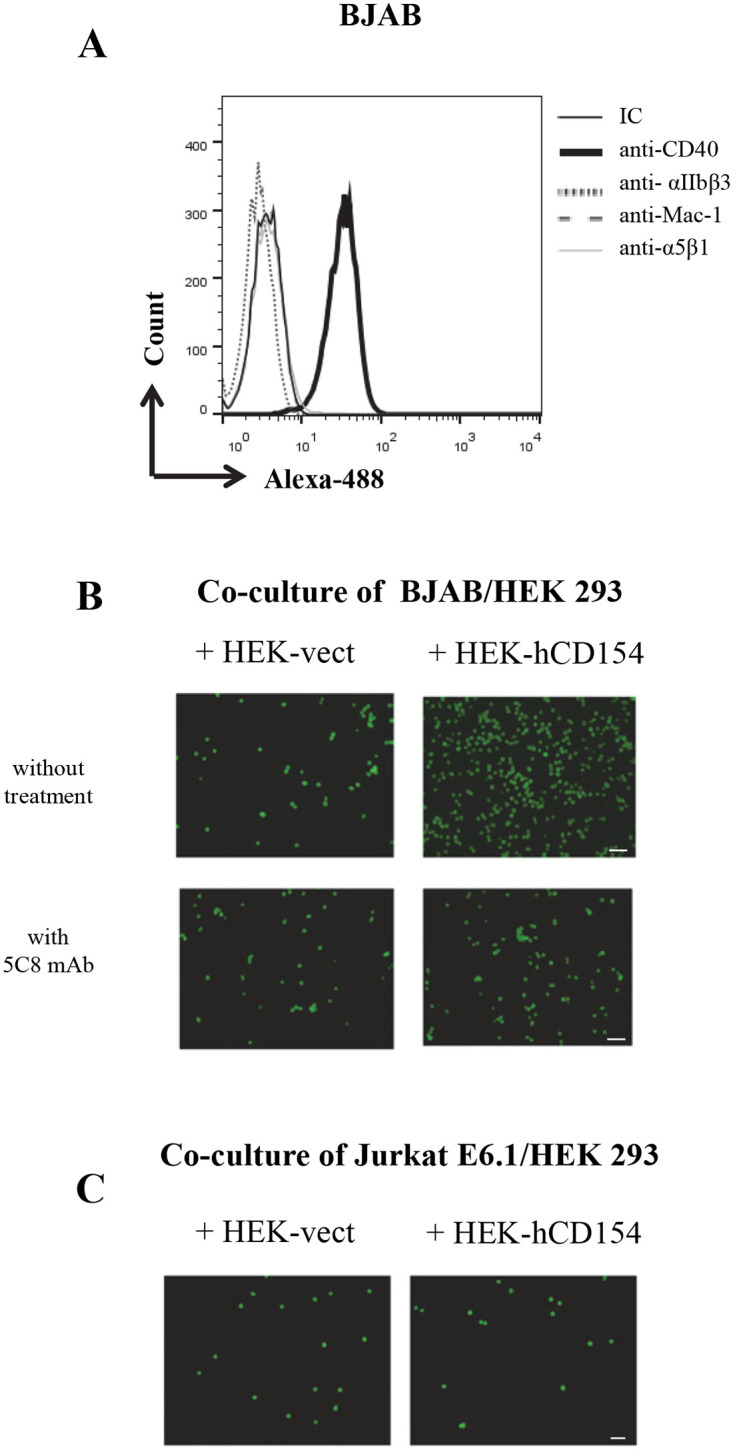
Jurkat E6.1 cells do not adhere to CD154-transfected HEK 293. (A) Expression of α5β1, CD40, Mac-1 and αIIbβ3 receptors on BJAB cells. BJAB cells were incubated with anti-α5β1 (JBS5 and B44), anti-CD40 (G28.5), anti-Mac-1 (ICRF44) and anti-αIIbβ3 (A2A9/6) mAbs or the corresponding isotype IgGs followed by goat anti-mouse IgG-Alexa488 staining and evaluated for expression of the corresponding molecule on cell surface by FACS analysis. *(B*, *C) Adherence of Jurkat E6*.*1 or BJAB cells to CD154-transfected HEK 293*. *(B)* BJAB cells were labelled with CFSE and treated or not with the anti-CD154 mAb, 5C8 before being co-cultured with HEK 293-vect. or with CD154-transfected HEK 293 (HEK 293-hCD154) for 5min at 37°C. Scale bar = 100μm. *(C)* Jurkat E6.1-NT cells were labelled with CFSE then co-cultured with HEK 293-vect. or with HEK 293-hCD154 for 5min at 37°C. The adherence of Jurkat E6.1 or BJAB cells to HEK293-hCD154 was analyzed by fluorescence microscopy. Scale bar in *(B)* = 100μm and in *(C)* = 20μm. Results represent mean values ±SD and are representative of at least 5 independent experiments.

The failure of membrane-bound CD154 expressed on HEK 293 cells to affect the cell death response can be due to the lack of interaction between the membrane-bound CD154 and α5β1. To verify this hypothesis, Jurkat E6.1 were labelled with CFSE and then added to CD154-transfected-HEK 293 cells adhered to 96 well plates. As a positive control, BJAB cells expressing CD40 on their surface (Fig 5A) were also labelled with CFSE and added to CD154-transfected-HEK 293 adhered on 96 well plates. Following a 5 min co-culture, wells were washed to remove non-adherent Jurkat E6.1 or BJAB cells, and cell/cell interactions mediated by CD154/α5β1 or by CD154/CD40 was analyzed by fluorescence microscopy. As shown in Fig 5B, CFSE-labelled BJAB cells, the CD40-positive cells, bound well to CD154-transfected HEK cells (Fig 5B upper panels). The latter cell/cell interaction was strongly inhibited by the treatment with the anti-CD154 mAb, 5C8, known to interfere with the binding of CD154 with CD40 (Fig 5B, lower panels). In contrast, CFSE-labelled Jurkat E6.1 cells, the α5β1-positive cells, did not interact with the CD154-HEK 293 (Fig 5C). These data further confirm that membrane-bound CD154 is not capable of interacting with the α5β1 integrin on cell surface as it does with CD40.

### 5-Membrane-bound CD154 expressed on Jurkat E6.1 T cells inhibits their Fas, TRAIL and TNF-α-mediated cell death

**Fig 6:**
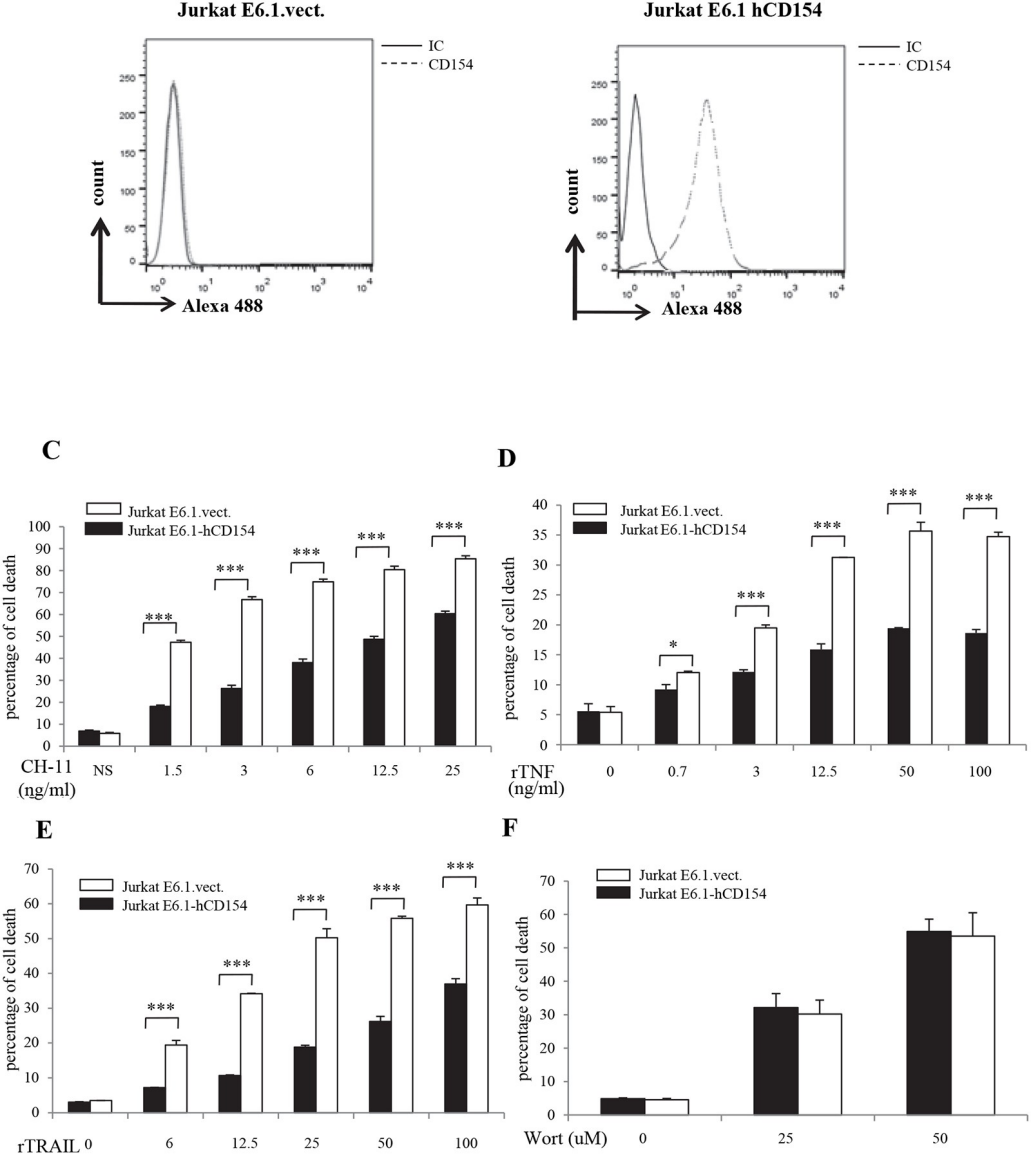
CD154 expression on the surface of Jurkat E6.1 cells inhibits their Fas-, TRAIL- and TNF-mediated death response. *(A*, *B)* Jurkat E6.1 cells were stably transfected with human CD154 (Jurkat E6.1-hCD154) or empty vector (Jurkat E6.1-vect.). Cells were then incubated with anti-CD154 (C4) mAbs or the corresponding isotype IgGs followed by goat anti-mouse IgG-Alexa488 staining and evaluated for expression of CD154 on cell surface by FACS analysis. *(C*, *D*, *E) Cell death response of CD154-transfected-Jurkat T cells stimulated with Fas ligand*, *TRAIL or TNF-α*. Jurkat E6.1-hCD154 and Jurkat E6.1-vect. T cells were stimulated with anti-Fas *(C)*, rTNF-α *(D)* or rTRAIL *(E)* for 18 hours at 37°C and assessed for their cell death response by FACS analysis as the percentage of death obtained by propidium iodide staining. *(F) Cell death response of CD154-transfected-Jurkat T cells stimulated with a survival inhibitor*. Jurkat E6.1-hCD154 and Jurkat E6.1-vect. T cells were stimulated with Wortmannin for18 hours at 37°C and assessed for their cell death response by FACS analysis. Results represent mean values ±SD and are representative of at least 5 independent experiments (** p < 0.*01*, *** p < 0.*001*).

Membrane-bound ligand (s) can interact with its membrane-bound receptor (s) in two different manners: 1) Trans interaction, when the ligand and its receptor are expressed in two different cells; and 2) Cis interaction, when both ligand and its receptor are expressed on the same cell [42]. Based on the above observation, we then investigated the possible interaction of membrane-bound-CD154 and -α5β1 in a cis manner. For this purpose, we used Jurkat E6.1 cells transfected with CD154 and evaluated their death response as compared to empty vector-transfected CD154-negative Jurkat cells. In the first set of experiments, we demonstrated the expression of CD154 on the surface of these cells by flow cytometry (Fig 6A and 6B). Our results confirm that CD154-transfected Jurkat E6.1 cells exhibit CD154 expression on their surface as compared to absence of the molecule from the surface of empty vector-transfected cells.

Next, we evaluated the apoptotic response of CD154-expressing Jurkat E6.1 cells to the various death factors mentioned above in comparison to that of empty vector-transfected Jurkat E6.1 cells lacking CD154 expression. Our results show that the stimulation with different concentrations of Fas induced a downregulated death response in CD154-expresing-Jurkat E6.1 cells as compared to CD154-negative cells (Fig 6C), and similar death patterns were observed for rTNF-α (Fig 6D) as well as rTRAIL (Fig 6E). Indeed, JurkatE6.1 cells transfected with CD154 were less sensitive to the apoptotic effect of Fas ligand, TRAIL and TNF-α than empty vector-transfected Jurkat E6.1 cells. These findings indicate that the CD154 expressed on the surface of Jurkat E6.1 cells was able to decrease Fas-, TNF-α-, and TRAIL-mediated death. To determine if the role of CD154 could be generalized to other forms of apoptosis, we examined its effect on apoptosis induced by the potent PI-3K inhibitor, Wortmannin [43, 44]. As shown in Fig 6F, empty vector-transfected as well as CD154-transfected Jurkat E6.1 cells exhibited similar apoptotic responses upon their stimulation with increasing concentrations of Wortmannin, indicating that CD154 does not abrogate the death response of T cells against a PI-3K inhibitor and that its effect observed above is not a non-specific effect on apoptosis in general.

Together, these results demonstrate that, similarly to the soluble form of CD154, the membrane-bound CD154 by interacting with its receptor, the α5β1 integrin, both expressed on the surface of the same cell, was capable of inhibiting apoptosis induced by various death receptors.

### 6-Membrane-bound CD154 binds to membrane-bound α5β1 in a cis manner

**Fig 7:**
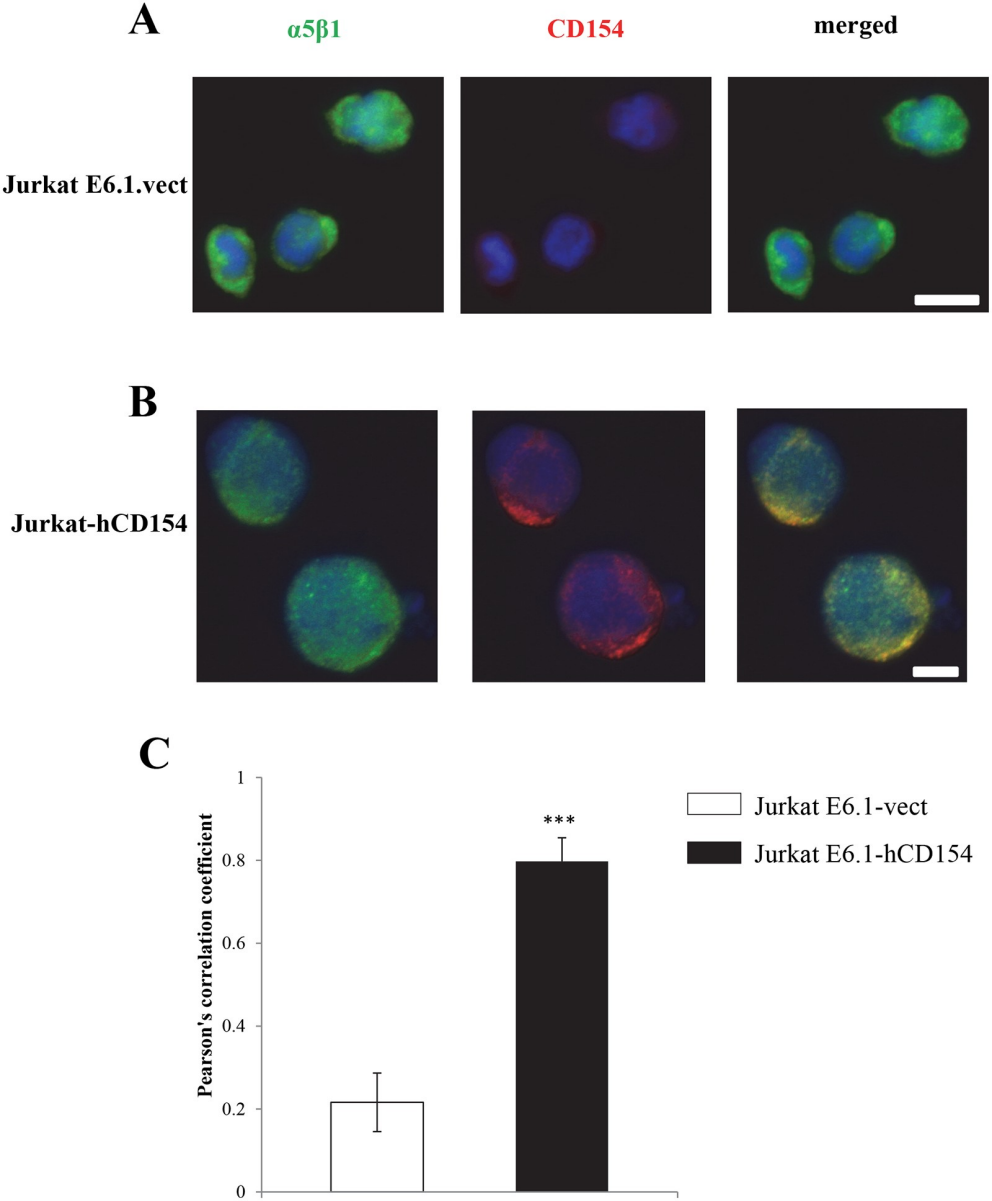
Cis association between membrane-bound CD154 and α5β1 on the surface of the same cell. *(A*, *B)* Jurkat E6.1-vect. *(A)* and Jurkat E6.5-hCD154 *(B)* cells were fixed in PFA and permeabilized in Triton before the addition of sCD40-Fc-biotinylated followed by streptavidin PE, to mark CD154 molecules in red and JBS5 followed by GAM-Alexa488 to mark the α5β1 integrin in green. DNA was counterstained with Hoechst dye. Images were recorded with a microscope (ApoTome.2, Observer. Z1; Carl Zeiss, Inc.) and driven by AxioVision LE software (Carl Zeiss, Inc.). *(C)* Quantification of co-localization sites. Co-localization between the integrin (red) and CD154 (green) was represented as Pearson’s correlation coefficient and measured in individual Jurkat cells. Scale bars in *(A*, *B)* = 10μm. Results represent mean values ±SD and are representative of 3 independent experiments (*** p < 0.*001*).

We demonstrated above that membrane-bound CD154 expressed on the surface of CD154-transfected Jurkat E6.1 cells interacting with α5β1 expressed on the same cell surface, inhibited their apoptotic responses while the membrane-bound form of CD154 expressed on another cell interacting with the α5β1 integrin of Jurkat E6.1 cells did not affect the death response of these latter. Thus, we highly suspected that the membrane-bound form of CD154 is only capable of interacting with α5β1 on cell surface in a cis-type of interaction. To confirm the cis-type of interaction between CD154 and the α5β1 integrin, we undertook a double immunofluorescence staining of these molecules on cell surface. For this purpose, we used empty vector-transfected Jurkat E6.1 cells as α5β1 positive/CD154 negative cells and CD154-transfected Jurkat E6.1 cells as α5β1 positive/CD154 positive cells. Subsequently, we localized the CD154 and α5β1 molecules using specific antibodies as described in the Materials and Methods section. Our data show that empty vector-transfected Jurkat E6.1 only exhibited staining for α5β1 on cell surface (Fig 7A), while CD154-transfected Jurkat cells showed staining for α5β1 (Fig 7B, left panel), for CD154 (Fig 7B, middle panel) and a visible co-localization of both molecules on cell surface (Fig 7B, right panel). Upon merging the red fluorescence (that of α5β1) and the green one (that of CD154), we quantified the area of co-localization measured in individual Jurkat E6.1 cells, represented as Pearson’s correlation coefficient. Our quantification results confirm the cis association of α5β1 and CD154 when both are expressed on the surface of the same cell (in CD154-transfected Jurkat E6.1, Fig 7C). Such co-localization of CD154 and α5β1 on cell surface of the same cell highly favours the possibility of a functional cis interaction between both molecules.

Taken together, our data confirm that the membrane-bound form of CD154 is capable of interacting with α5β1 integrin on cell surface in a cis-type of interaction. The biological significance of this phenomenon might be to sequester the available CD154 on the surface of activated T cells with the α5β1 integrin enhancing the T cell survival while limiting the accessibility to trans binding including its binding to the other receptors on other target cells, providing a certain form of immunological balance.

## Discussion

The role of CD154 in immune responses has long been established. By interacting with its classical receptor CD40, CD154 has been implicated in humoral and cell-mediated immunity, by inducing activation of several types of immune cells as well as affecting their proliferation and apoptosis processes, contributing as such to the development of numerous inflammatory and autoimmune diseases. The more recent identification of additional receptors for CD154 solicited the interest in digging deeper into the role of CD154 in immune responses and their related pathologies. Interestingly, our group has previously shown that the CD154/α5β1 interaction inhibits Fas-induced death in T cell lines [29], strongly suggesting that the CD154/α5β1 interaction enhances the survival and persistence of T cells. In the current study, we wanted to further define the role of CD154 in T cell survival using not only T cell lines but also human primary T cells. Our data show that the sCD154 was capable of inhibiting apoptosis induced not only by Fas-Ligand, but also by other death signals, namely TRAIL and TNF-α. This is likely due to the capacity of CD154 to inhibit caspase-8 activation downstream of death receptors. Indeed, we have previously reported that CD154 inhibited Fas-induced apoptosis of Jurkat T cells by inhibiting the cleavage and activation of caspase-8 [29]. Interestingly, the protective effect of CD154 is also seen in human primary T cells suggesting that α5β1 integrin engagement with CD154 could be a critical pathway that contributes to the survival and persistence of effector T cells in inflammatory diseases. Along these lines, the effect seems to be more important for CD4^+^ than CD8^+^ T cells. Mechanisms accounting for these differences are not clear but could be due to the higher levels of 〈5^®^1 integrin expressed by CD4^+^ and to the possibility that α5β1 integrin is connected to different downstream signalling molecules in the two T cell populations.

Our results also show that Jurkat E6.1 cells and human T cells exhibit a surface expression of the α5β1 integrin in its inactive form, a form shown to be the only state of the molecule capable of binding to and interacting with CD154 [23], and do not exhibit any expression of CD40 on cell surface. These results are not surprising, given the wide expression profile of α5β1 on the surface of numerous cell types [11, 45], including T cells [46]. Inducing apoptosis of T cells is a mean by which our immune system is regulating its responses [47], trying to decrease the load of T cells in related conditions such as inflammatory and autoimmune diseases [11, 47]. Thus, by inhibiting this process, the CD154/α5β1 dyad is contributing to T cell survival and persistence in inflammatory conditions [34–36]. Many inflammatory diseases are characterized by an elevated number of T cells such as RA [48], multiple sclerosis [49, 50], celiac disease [51], and even SLE [52]. Interestingly, T lymphocytes in autoimmune diseases were shown to express aberrant amounts of β1 integrins [53, 54]. Not only the receptor, but also the ligand is increased in autoimmune/inflammatory diseases [8, 9]. More importantly in this matter is the fact that not only membrane-bound but also sCD154 is elevated in autoimmune/inflammatory diseases, increasing the chance of a functional CD154/α5β1 interaction at least with respect to the inhibition of T cell death, since, as described by our data here, sCD154 is more potent in interacting with the α5β1 integrin while the membrane-bound form of the molecule was not capable of such interaction in human T cells. Another important point with respect to the survival-enhancing effect of sCD154 in T cells is its increased importance in CD4^+^ T cells, the T cell population usually of high significance in autoimmune/inflammatory diseases [55–57]. Therefore, our results and previously reported findings highly suggest that the CD154/α5β1 binding is highly implicated in autoimmune/inflammatory responses, more specifically in inhibiting T cell death enhancing as such their survival and persistence in these diseases.

Going back to our data herein and our previous studies [29], showing that the soluble form of CD154 was capable of inhibiting apoptosis induced by the death signals Fas, TRAIL as well as TNF-α, we were solicited to determine if the membrane-bound form of CD154 was also capable of eliciting similar effects with regard to cell death induced by the above-mentioned apoptotic factors. In spite the fact that both membrane-bound and soluble CD154 are biologically active [3], the interaction of the former with its receptor implicates a more complex form of binding given the transmembrane structure of the membrane-bound CD154 and its anchorage in the plasma membrane. Both soluble and membrane-bound CD154 were shown capable of interacting with CD40 [58–60]. As to the interaction of CD154 with its newly identified receptors, the 〈IIb^®^3 [25, 61], and 〈M^®^2 integrins [22], all data documented with respect to the induced intracellular signaling events and the subsequent biological responses describes the binding of the soluble form of CD154 with the integrins expressed on cell surface. In our current study, our data show that Jurkat E6.1 cells that express α5β1 on their surface co-cultured with CD154-transfected HEK 293 exhibited similar death levels as Jurkat E6.1 cells co-cultured with CD154-negative HEK, indicating that membrane-bound CD154 is not capable of interacting with the α5β1.

Interestingly, in the matter of membrane-bound ligands and their receptors, it is well established that their interaction could be undertaken in two different manners: 1) a trans interaction, when the ligand and its receptor are expressed in two different cells; and 2) cis interaction, when both ligand and its receptor are expressed on the same cell [42]. This is particularly of interest in the case of the CD154/α5β1 dyad, as both the ligand and the receptor could be expressed on the surface of the same cell [42]. Different biological responses could even be issued from cis- as compared to the trans-type of binding in the ligand/receptor complex [42]. Indeed, the cis- versus trans- type of interaction on cell membranes have been demonstrated for many cell surface molecules, among the first was the CD22 on the surface of B cells [62]. Such molecule binds sialic acid on B cell surface, more importantly in a cis-fashion, owing to the high concentration of sialic acid in the B cell membrane [63]. Importantly, CD22 is associated with BCR and its cis-binding to sialic acid herein is believed to halt hyperstimulation of the BCR [64]. Also, ligand/receptor dyads involving protein-protein interactions were shown to exhibit a cis- and a trans-type of interaction. The Natural Killer (NK) cell inhibitory receptors specific for MHC-I molecules interact with these latter expressed on target cells (trans-fashion) as well as with MHC-I expressed on NK cells themselves (cis-interaction) [65]. An example here is the Ly49A binding to the H-2D^d^ allele, most of which is undertaken is a cis-manner, controlling the number of receptors available for trans binding and the stability of the binding [66, 67]. Interestingly, more recent studies by Saggu et al., have shown that the CD18 integrin, Mac-1, on the surface of neutrophils, is capable of cis-binding the FcγRIIA receptors, decreasing their affinity to IgG complexes and thus reducing the recruitment of neutrophils to the immune site [68]. Indeed, authors have demonstrated that silencing of CD18 in mice using siRNA or knocking out the corresponding gene leads to an accumulation of neutrophils in kidneys upon mice injection with a nephrotoxic serum [68]. Thus, the cis-interaction of Mac-1 and FcγRIIA is a mean by which the immune system regulates neutrophil accumulation, and a disruption of such process could have significant implications in inflammatory and autoimmune diseases. Other examples of receptor/ligand dyad interacting in a cis fashion exist and are reviewed in [42, 69]. In investigating the cis versus trans interaction of the CD154 with the α5β1 integrin, we demonstrated that membrane-bound CD154 interacting with the α5β1 integrin, both expressed on the surface of the same cell, is capable of abolishing the apoptosis of this cell induced by Fas ligand, TNF-α or TRAIL. Both molecules are also shown to co-localize when expressed on the surface of the same cell. With respect to this latter point, it has been previously reported that co-localization of two molecules increases the possibility of a functional interaction between them [70]. Our data thus indicate that membrane-bound CD154 is not capable of binding the α5β1 in a trans-type of interaction, and is only capable of interacting with α5β1 on cell surface in a cis-type of interaction. The biological significance of this phenomenon might be to sequester the available CD154 on the surface of activated T cells with the α5β1 integrin enhancing the T cell survival while limiting the accessibility to trans-binding including its binding to the other receptors on other target cells, providing a certain form of immunological balance.

In summary, our study further outlines the immune/inflammatory signature of CD154 and adds to the list of roles this molecule can play in the pathogenesis of autoimmune diseases. A special attention was given to the newly discovered, and thus less visited CD154 receptors, namely the α5β1 integrin. Our study highlights the biological significance of the CD154/α5β1 dyad more specifically in enhancing the survival and persistence of T cells, a phenomenon of high importance in inflammatory and autoimmune conditions such as RA and SLE.

## Abbreviations

sCD154: soluble CD154
TNF: tumor necrosis factor
TRAIL: TNF-related apoptosis-inducing ligand
